# Supplementary material for: Research on trust mechanism of supply chain finance under Industrial Internet embedded with blockchain
Source: PLoS One. 2024 Jun 24;19(6):e0299011. doi: 10.1371/journal.pone.0299011 (PMC11195946; doi:10.1371/journal.pone.0299011)
Supplement: Attachment — Submitted filename: Response to Reviewer 2 Comments.docx [file pone.0299011.s003.docx]

Dear Reviewer,

We deeply appreciate the time and effort you've dedicated to reviewing our manuscript titled " Research on trust mechanism of supply chain finance under Industrial Internet embedded with blockchain". Your comments provide valuable insights which have enabled us to refine our paper. Here's a summary of our revisions in response to your feedback:

**Point 1:** 1. Is the empirical method used experimental? or?

**Response 1:** We are very sorry to cause you any doubt in this respect. In order to explain the method used in this paper and its function, we have re-described it in the introduction, which is detailed as follows.

This paper comprehensively explores the applicability and role of Industrial Internet technology and blockchain technology in supporting supply chain finance activities, and constructs a supply chain finance platform architecture that integrates blockchain technology and industrial internet. By utilizing the distributed data storage, dynamic transaction monitoring, and massive data modeling and analysis technologies of the Industrial Internet platform and blockchain, a global trust evaluation model that integrates Funk-SVD and information entropy is designed. Flexible and intelligent machine learning recommendation algorithm is used to generate a trust value that includes comprehensive trust information about financing companies. This aims to improve the accuracy of trust assessment for SMEs in supply chain, and alleviate the information asymmetry in supply chain finance. Finally, the proposed algorithm is subjected to performance tests using a publicly available dataset, confirming its superiority in processing large sample data. Based on the analysis of the test results, corresponding countermeasures are proposed.

**Point 2:** The data used is also briefly explained. Is it from https://snap.stanford.edu/data/soc-Epinions1.html?

**Response 2:** We thank you for your inquiry about the data source. Indeed, the data used in this study was derived from the dataset available at https://snap.stanford.edu/data/soc-Epinions1.html.

The Epinions dataset is derived from real data of the Epinions online review site, comprising 'ratings_data' and 'trust_data.' Epinions operates akin to an e-commerce site where users rate products, and this data is captured in 'ratings_data.' Other users who have not experienced the product can use these aggregated ratings as a reference for potential purchases. Additionally, Epinions features a 'trust' option, allowing users to specify those they trust, and the ratings from these trusted users are recorded in 'trust_data,' lending greater weight to their reviews. The unique trust network of Epinions has made it a classic dataset for researching trust mechanisms and rating behaviors on e-commerce social platforms, as well as other recommendation algorithms within e-commerce. Similarly, industrial Internet supply chain financial platforms also embody characteristics of supply chain business transaction networks and product-service interactions. Therefore, leveraging the trust and rating data from the Epinions dataset could be instrumental in testing trust evaluation algorithms on these platforms.

**Point 3:** It would be better to add graphs, descriptive statistics and hypothesis testing, for example a difference test using the t-test. In conducting performance analysis, this research only displays data and does not explain the meaning of the parameters in the table. For example, table 4, what is meant by RMES? What is meant by Root Mean Square Error (RMSE)?

**Response 3:** Thank you very much for your suggestion. In this paper, we design a global trust evaluation model which integrates Funk-SVD and information entropy. It aims to improve the accuracy of trust assessment in supply chain finance and alleviate the information asymmetry. Actually, the model in this paper focuses on performance testing and validation of the proposed algorithm, so there is no difference test. Thank you for the suggestion on the research method, which may become one of the directions for our future research on supply chain finance trust assessment. And, when performing performance analysis, this study really only presents the data and does not explain the meaning of the parameters in the table. Now, we have added specific explanations at the relevant section of the paper.

To facilitate expression, the number 1 is used to represent the parameter situation before implementing dimensionality reduction and, and the number 2 is used to represent the parameter situation after implementing dimensionality reduction and focusing. RMES represents the root-mean-square error of trust ratings, which is the square root of the sum of the squared differences between the actual values and the predicted values, divided by the number of predictions.

The "step" refers to the number of iterations and "α" refers to the step size during each optimization step in the process of fitting the initial model to the optimal model. "RMSE1" represents the error after the first iteration of the evaluation matrix. "RMSE2" represents the error value between the new rating matrix after i iterations with the original matrix, after i iterations with the original matrix. "TIME" refers to the program running time needed to converge the loss function to the optimal value through iterations.

**We are grateful for your comprehensive feedback, and we hope that the revisions made will elevate the quality and relevance of our research. We look forward to your further comments and suggestions.**

**Warm regards,**

**Yingmei Jiang**

**Jinhua Polytechnic**
